# Antinociceptive principle from *Curcuma aeruginosa*

**DOI:** 10.1186/s12906-015-0720-6

**Published:** 2015-06-20

**Authors:** Chowdhury Faiz Hossain, Mohammad Al-Amin, Abu Sadat Md. Sayem, Ismail Hossain Siragee, Asif Mahmud Tunan, Fahima Hassan, Md. Mohiuddin Kabir, Gazi Nurun Nahar Sultana

**Affiliations:** Present address: Department of Pharmacy, East West University, Plot no-A/2, Jahurul Islam Avenue, Jahurul Islam City, Aftabnagar Dhaka-1212 Bangladesh; Department of Genetic Engineering and Biotechnology, East West University, Plot no-A/2, Jahurul Islam City, Aftabnagar Dhaka-1212 Bangladesh; Centre for Advanced Research in Sciences (CARS), University of Dhaka, Dhaka, 1000 Bangladesh

**Keywords:** Zingiberaceae, Antinociceptive activity, Germacrone (**1**), Cyclic sesquiterpene, Acetic acid, Formalin

## Abstract

**Background:**

The rhizome of *Curcuma aeruginosa* Roxb (Zingiberaceae) has been used as a traditional folk medicine for the treatment of rheumatic disorders in Bangladesh. The aim of the current study was the bioassay-guided isolation and purification of an antinociceptive principle from the methanol extract of *C. aeruginosa* rhizomes*.*

**Methods:**

The antinociceptive activity was determined using acetic acid induced writhing and formalin induced licking in the Swiss albino mice to investigate central and peripheral antinociceptive principle of *C. aeruginosa* rhizomes. Vacuum Liquid Chromatography (VLC) and open column chromatography were used for separation. Crystallization was used for the purification of the isolated compound germacrone (**1**). Diclofenac (10 mg/kg) and aspirin (100 mg/kg) were used as positive control and 5 % carboxymethyl cellulose (CMC) in distilled water (10 ml/kg) for negative control were used in the acetic acid induced writhing and formalin induced licking methods.

**Results:**

The methanol extract exhibited 37.50 and 45.31 % inhibition of writhing; 33.27 and 38.13 % inhibition of licking in the first phase and 69.72, 73.71 % inhibition of licking in the second phase at doses of 200 and 400 mg/kg, respectively. VLC of the extract yielded five fractions (Fr. 1 to Fr. 5). Fr. 1 exhibited 33.98 % inhibition that was comparably higher than other fractions (Fr. 2 to Fr. 5) at a dose of 100 mg/kg. Column chromatography of Fr. 1 generated five fractions (SF. 1 to SF. 5). Fraction SF.3 exhibited 46.88 % inhibition that was most potent among the other fractions at a dose of 50 mg/kg. Crystallization of the fraction SF.3 yielded germacrone **(1)**, a cyclic sesquiterpene. Germacrone (**1**) showed 22.66, 34.77 and 51.17 % inhibition of writhing at doses of 10, 20 and 40 mg/kg, respectively; 30.43 and 37.53 % inhibition in the initial phase and 32.27 and 60.96 % inhibition in the second phase of licking at doses of 200 and 400 mg/kg, respectively.

**Conclusion:**

Germacrone (**1**) showed a potent activity in both writhing and licking methods that indicates the compound as a central and peripheral antinociceptive principle of *C. aeruginosa* rhizomes with possible anti-inflammatory activity.

## Background

As part of our continuing bioassay-guided isolation and purification of the active principles from traditional medicines [1–3], we investigated one of the most useful Bangladeshi medicinal plants, *Curcuma aeruginosa* Roxb (Zingiberaceae; Bangladeshi local name:- Kathali holud). *C. aeruginosa* is one of the most prominently used medicinal plants in Bangladesh, India, Myanmar, Indonesia, Malaysia, and Thailand. The rhizome is very popular as a folk medicine in Bangladesh for the treatment of pain and inflammation associated with rheumatic diseases. In addition, the different parts of this plant are also commonly used as traditional medicines for the treatment of uterine pain, uterine inflammation and various gastrointestinal disorders such as diarrhea and colic [4]. Pharmacological activities such as antinociceptive, antipyretic, anti-inflammatory [5], antimicrobial [6, 7], antioxidant [8, 9] and anti-androgenic properties [10] have been previously reported. Germacrone, furanodiene, curcumenol, zedoarol, zedoarondiol, zedoalactone A, zedoalactone B, isocurcumenol, and isofuranodiene are the major chemical constituents isolated from the rhizomes of *C. aeruginosa* [4, 11]. Zedoarol, curzerenone, furangermenone, furanodienone, curcumenol, 1, 8-cineol and camphorare are the major volatile compounds of *C. aeruginosa* [12]. Although, the antinociceptive activity of the methanol and chloroform extracts of the rhizome of *C. aeruginosa* [5] and the anti-inflammatory activity of germacrone (**1**) [13] have been reported, the active principle that causes the antinociceptive activity of *C. aeruginosa* rhizome is still unknown and needs to be identified.

On the basis of the use of a plant as the folk and traditional medicine as well as the pharmacological properties reported in the literature, the present study was carried out to investigate the antinociceptive activity of the MeOH extract of *C. aeruginosa* rhizome and the active principle it contained through bio-assay guided fractionations.

## Methods

### Drugs and chemicals

Organic solvents were obtained from Merck, Germany; Diclofenac was purchased from Beximco Pharma, Bangladesh; Aspirin was purchased from Square Pharmaceutical Ltd, Bangladesh; Thin Layer Chromatography (TLC) was run on Merck TLC plates precoated with Si_60_ F_254_ with visualization by spraying with 10 % H_2_SO_4_ in MeOH followed by heating. VLC was done using Silica gel 60 (0.040–0.005 mm), Merck, Germany. Open column chromatography was done by using Silica gel 60 (0.063–0.020 mm), Merck, Germany. The IR Spectrum was obtained using a Shimadzu IR Prestige-2 FT-IR while the ^1^H-NMR spectra were recorded on an ultra shield Bruker DPX 400 spectrometer. The NMR spectra were recorded running gradients and using residual solvent peak (*δ* 7.25 s for ^1^H-NMR) as an internal reference.

### Plant materials and Extraction

*C. aeruginosa* rhizomes were collected from Panchagarh, Rangpur Division of Bangladesh, in December 23, 2011 and their identity was confirmed by the National Herbarium of Bangladesh, Chiriakhana Road, Mirpur-1, Dhaka-1216, Bangladesh, where a voucher specimen has been deposited (Accession Number 37514). After collection, the rhizomes were washed with water, cut into pieces and sun-dried because aqueous suspensions of sun-dried powder is used in the folk and traditional medicinal preparation. Then, dried rhizomes were powdered by using a Spices Mill. Dried powdered rhizomes (1.5 kg) were extracted with MeOH at room temperature (4000 ml × 72 h × 3 times). The extracting solvent was filtered, and the filtrate was concentrated *in vaccuo* by a rotary evaporator (45 °C) to get the crude MeOH extract (195.0 g, yield: 13 % from dried powder).

### Separation of the MeOH extract

**Fig. 1:**
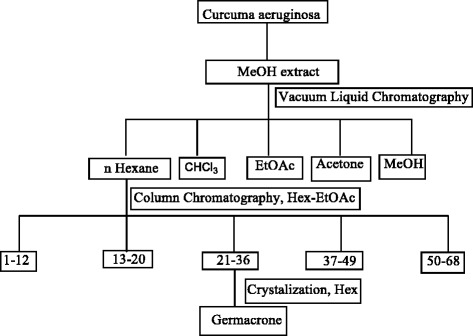
Isolation and purification of germacrone (1) from *curcuma aeruginosa* rhizome

The crude MeOH extract (175 gm) was subjected to a silica gel (120 gm) VLC and eluted with *n*-hexane (5 L), chloroform (5 L) , EtOAc (5 L), acetone (5 L) and MeOH (5 L) to obtain Fr. 1 (48 gm), Fr. 2 (29 gm), Fr. 3 (20 gm), Fr. 4(43 gm) and Fr. 5 (17 gm), respectively. On the basis of the analgesic activity, Fr. 1 (48 gm) was subjected to silica gel (300 gm) open column chromatography and eluted with *n*-hexane-EtOAc step gradients. Following this, 68 fractions of 200 ml each were collected as follows: 1–48 (*n*-hexane), 49–54 (*n*-hexane- EtOAc, 9:1), 55–58 (*n*-hexane-EtOAc, 8:2), 59–62 (*n*-hexane-EtOAc, 5:5) and 63–68 (EtOAc). On the basis of the analytical TLC, these fractions were then pooled into five new fractions (SF. 1 to SF. 5) as follows: SF. 1 (1–12, 7gm), SF. 2 (13–20, 5.0 gm), SF. 3 (21–36, 3 gm), SF. 4 (37–49, 6 gm), SF. 5 (50–68, 16 gm). The analgesic activity of SF. 1 to SF. 5 were evaluated and the most potent fraction (SF. 3, 3.0 gm) was crystallized from *n*-hexane to get colorless rod shape crystals of germacrone (2 gm, yield: 1.14 %). The schematic diagram of the bio-assay guided isolation and purification of germacrone (**1)** from the MeOH extract are shown (Fig. 1).

**Fig. 2:**
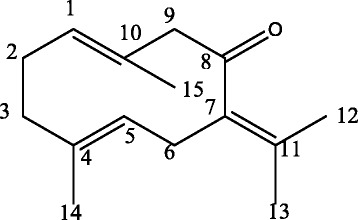
Germacrone (1)

Compound **1**(Germacrone): R_f_ value on Si-gel TLC with *n*-hexane: EtOAc (9.5:0.5) as mobile phase is 0.55. IRν_max_ (KBr) cm^−1^: 1129, 1289, 1384, 1438, 1672, 2849, 2915, 2983. ^1^H-NMR (CDCl_3_) *δ* (ppm): 1.42 (3H, s, 14-H), 1.61 (3H, s, 15-H), 1.70 (3H, s, 13-H), 1.75 (3H, s, 12-H), 2.09 (3H, s, 2-H_b_, 3-H_a_, 3H_b_), 2.34 (1H, s, 2-H_a_), 2.83 (1H, d, *J* = 12.5 Hz., 6-H_b_), 2.93 (2H, d, *J* = 11.0 Hz., 6-H_a_, 9-H_a_), 3.39 (1H, d, *J* = 10.1 Hz., 9-H_b_), 4.69 (1H, d, *J* = 10.1 Hz., 5-H), 4.96 (1H, d, *J* = 11.5 Hz., 1-H). The chemical structure of germacrone (**1**) is shown ( Fig. 2).

### Animals

All Swiss albino mice of either sex, weighing 22–28 gm, obtained from the Animal Resource Division, International Center for Diarrhoeal Disease and Research, Bangladesh (ICDDR,B), were used throughout the experiments. All animals were kept in standard environmental conditions, had free access to standard food (ICDDR,B formulated) and water *ad libitum* and fasted 18 h prior to their use [14]. The animal experiments were carried out in accordance with the guiding principles (Directive 2010/63/EU of the European Parliament and of the Council of 22 September 2010) on the protection of animals used for scientific purposes and approved by the Departmental Animal Care and Use Committee of East West University.

### Acetic acid induced writhing

The antinociceptive activity of the samples was evaluated using acetic acid induced writhing in mice [15–18]. In this method, acetic acid was administered intraperitoneally (i.p.) in the experimental animals to create a painful sensation. As a positive control, diclofenac was used. The plant extract, VLC fractions, SF. 1 to SF. 5 and germacrone (**1)** were administered orally (200, 400 mg/kg body weight for the crude extract, 100 mg/kg for VLC fractions, 50 mg/kg for SF. 1 to SF. 5 and 10, 20, 40 mg/kg for germacrone) to the Swiss Albino mice after an overnight fast. Suspensions of all test samples were prepared with 5 % carboxymethyl cellulose (CMC) in H_2_O for oral administration. Test samples (MeOH extract, VLC fractions, SF. 1 to SF. 5 and germacrone) and a negative control (5 % CMC in H_2_O) were administered orally 30 min prior to intraperitoneal administration of a 0.7 % v/v acetic acid (100 %) solution (0.1 ml/10gm). Diclofenac was administered 15 min prior to acetic acid injection. Then, the animals were placed on an observation table. Each mouse of all groups was observed individually to count the number of writhing they made in 10 min commencing just 5 min after the intraperitoneal administration of the acetic acid solution. Full writhing was not always observed in the animals as sometimes the animals started to writhe but they did not continue. This incomplete writhing was considered as half-writhing. Accordingly, two half writhing were taken as one full writhing. The number of writhing in each treated group was compared to that of a control group in which diclofenac (10 mg/kg) was used as a reference (positive control).

### Formalin-induced hind paw licking

The experiment was performed with slight modification of the published methods [16, 18–21]. 5 % formalin solution (20 μl) prepared in 0.9 % normal saline was injected subcutaneously into the right hind paw of mice. The time (in seconds) spent on licking the injected paw was considered as an indication of the amount of pain. The first phase of the nociceptive response normally peaks at 5 min and the second phase 15–30 min after formalin injection, representing central and peripheral antinociceptive and anti-inflammatory pain responses, respectively. The mice (22–28 gm) were divided into groups and each group had five mice (n = 5). The mice were fasted for 24 h prior use but were allowed free access to water. The animals received 10 ml/kg of 5 % CMC in H_2_O as negative control, 200, 400 mg/kg of the MeOH extract, 100 mg/kg of VLC fractions and 20, 40 mg/kg of germacrone (**1**) for 1 h before being challenged with formalin, while 100 mg/kg of acetylsalicylic acid (aspirin) was used 30 min prior to formalin injection. All the tested drugs, the negative control, the MeOH extract, fractions, germacrone (**1)** and aspirin (positive control) were administrated orally by use of a feeding needle. Suspension of all test samples was prepared with 5 % carboxymethyl cellulose (CMC) in H_2_O for oral administration. The responses were measured for 5 min (first phase) and 15–30 min (second phase) after formalin injection.

### Statistical analysis

All data were expressed as mean ± SEM using one-sample *t*-test. Comparison of the analgesic activity in all groups was made using one-way ANOVA followed by Dunnett’s multiple comparisons test. The significance level was *p* < 0.05. All statistical analyses were conducted using SPSS (Version 17) software package for Windows (Chicago, IL, USA).

## Results and discussion

Acetic acid induced writhing is an useful method for investigating peripheral antinociceptive drug leads [17, 18]. Pain sensation associated with irritation of peritoneal cavity by intraperitoneal administration of acetic acid is characterized by abdominal contractions, movements of the body as a whole, twisting of the abdominal muscles, and a reduction in motor activity. Moreover, in this method, pain is generated by stimulating peripheral nociceptive neurons via endogenous mediators such as bradykinin, serotonin and capsaicin. Thus, the peripheral nociceptive response of abdominal writhing induced by acetic acid mainly involves the release of arachidonic acid metabolites via cyclooxygenase (COX), and prostaglandin (PGE2 and PGF2α) biosynthesis [18, 22, 23].

**Fig. 3:**
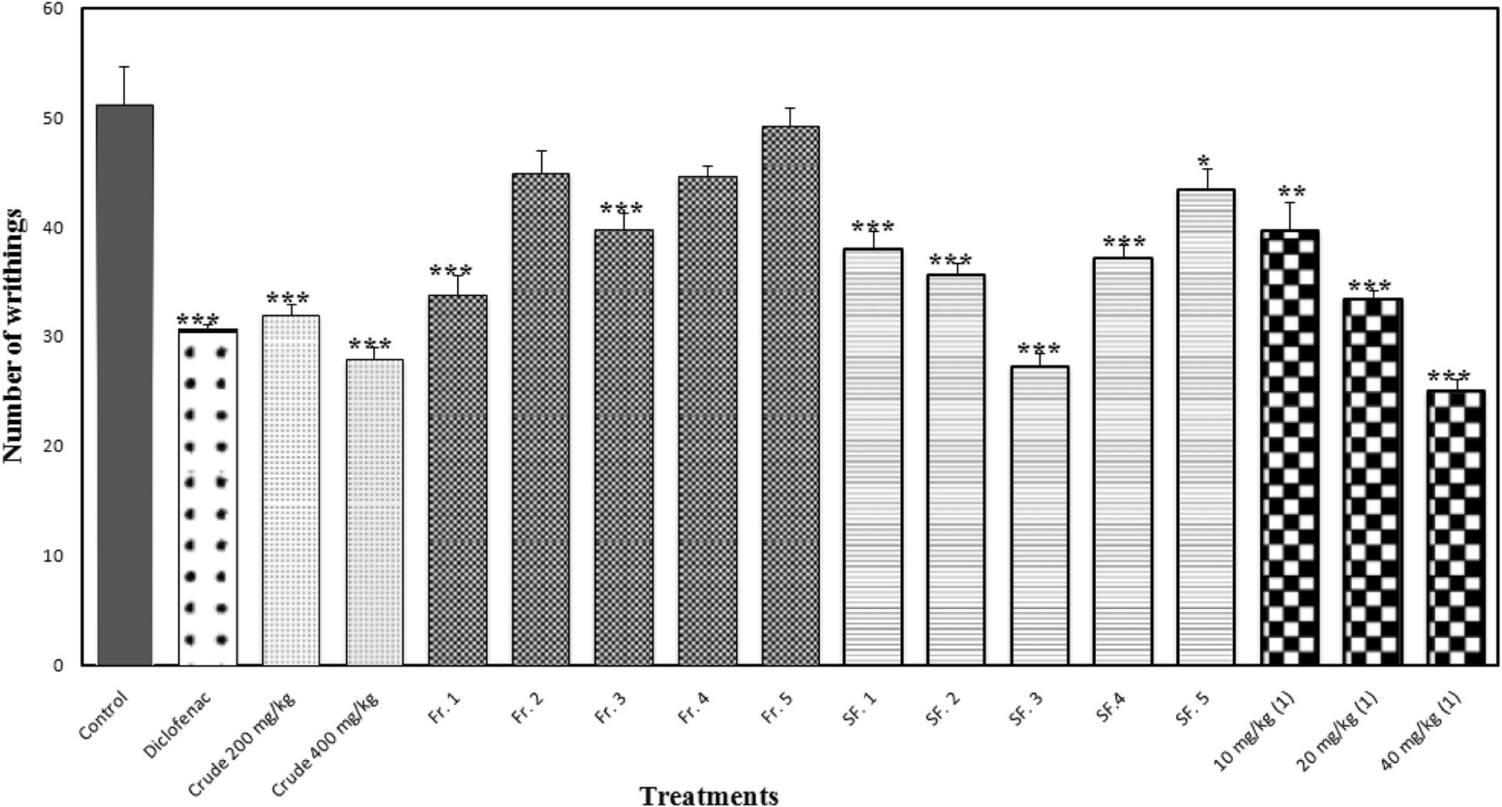
Effects of different doses of MeOH extract, VLC fractions, SF. 1 to SF. 5 and germacrone (1) on acetic acid induced writhing method in mice. Results are mean ± SEM for five mice. Statistical comparison was performed using ANOVA followed by Dunnett’s test. * *p* < 0.05, ** *p* < 0.001 and *** *p* < 0.001, when compared with control group

The MeOH extract demonstrated statistically significant peripheral antinociceptive activity compared to the reference drug, diclofenac, by reducing the number of acetic acid induced writhing in mice at doses of 200 and 400 mg/kg. Hence, the result supports the presumption that the extract may act by inhibiting prostaglandin biosynthesis [23]. The MeOH extract of *C. aeruginosa* in acetic acid induced writhing exhibited 37.50 % and 45.31 % inhibition (*p* < 0.001) at doses of 200 and 400 mg/kg, respectively (Fig. 3). The selection of doses for the extract and the fractions were kept far below the published toxicity study of the crude extract in the literature [5]. This result is an indicative for the peripheral antinociceptive activity of the *C. aeruginosa* extract.

The formalin induced licking is an useful method to distinguish between the central and peripheral antinociceptive action [18]. This method exhibits a biphasic nociceptive response described as the first and the second phases. The initial phase, neurogenic pain, is occurred by C-fiber activation produced by direct stimulation of nociceptive neurons; while the second phase, inflammatory pain, is caused by local tissue inflammation and also by functional changes in the dorsal horn of the spinal cord [22, 24]. The initial phase is occurred immediately after the injection of formalin while the second phase is exhibited between 20 and 30 min post-injection [22]. In addition, it is reported that substance P and bradykinin are participated for the response of the first phase while histamine, serotonin, prostaglandin and bradykinin are involved for the response of the second phase [25]. Therefore, the second phase is inhibited both by opioids and non-steroidal anti-inflammatory drug (NSAIDs) [24]. In this ways, substances that inhibit both phases are considered as central antinociceptive while the substances that inhibit second phase are considered as peripheral antinociceptive with possible effect on inflammation [22, 24].

**Fig. 4:**
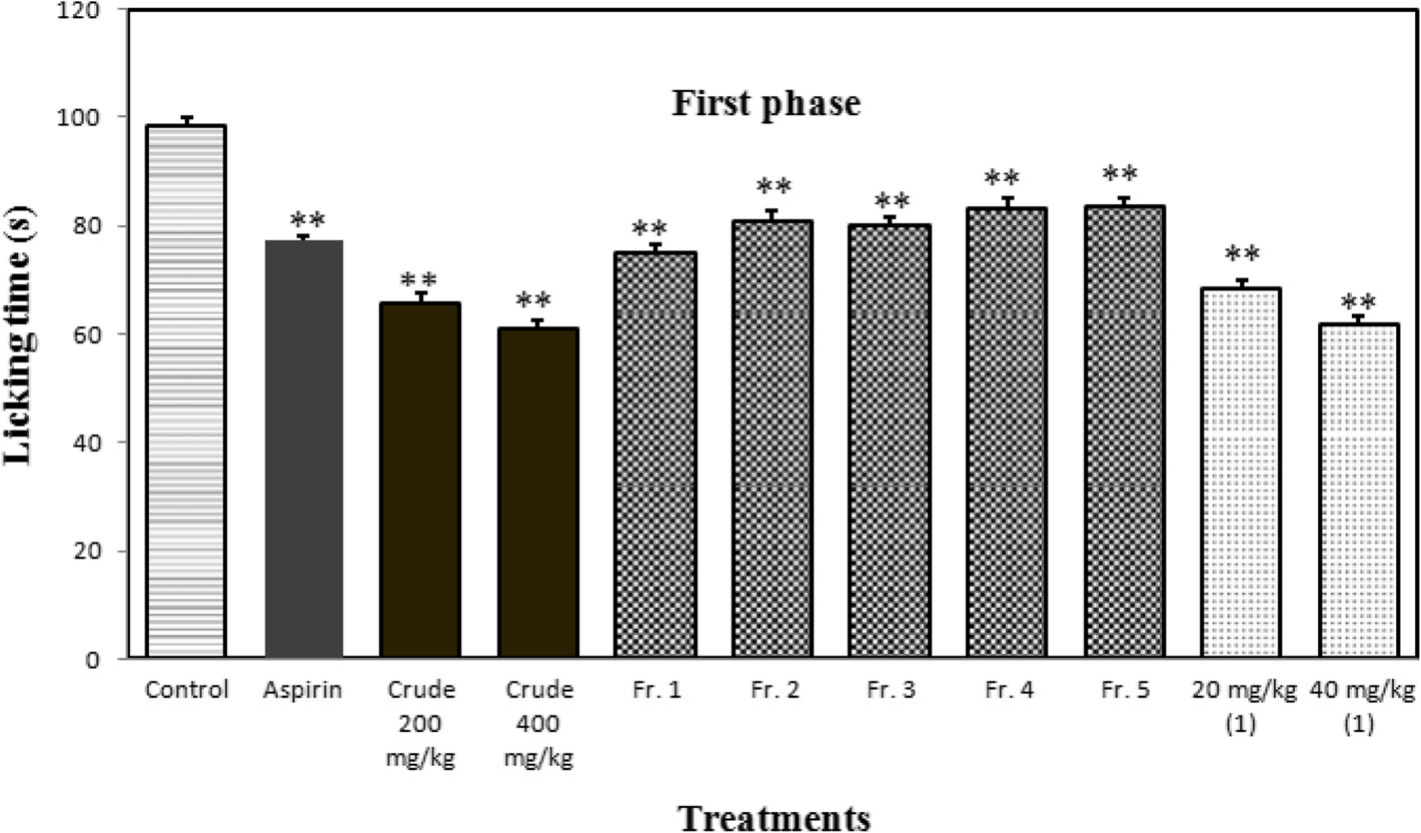
Effects of different doses of MeOH extract, VLC fractions and germacrone (1) on formalin induced licking in mice (First Phase). Results are mean ± SEM for five mice. Statistical comparison was performed using ANOVA followed by Dunnett’s test. * *p* < 0.05, ** *p* < 0.001, when compared with control group

**Fig. 5:**
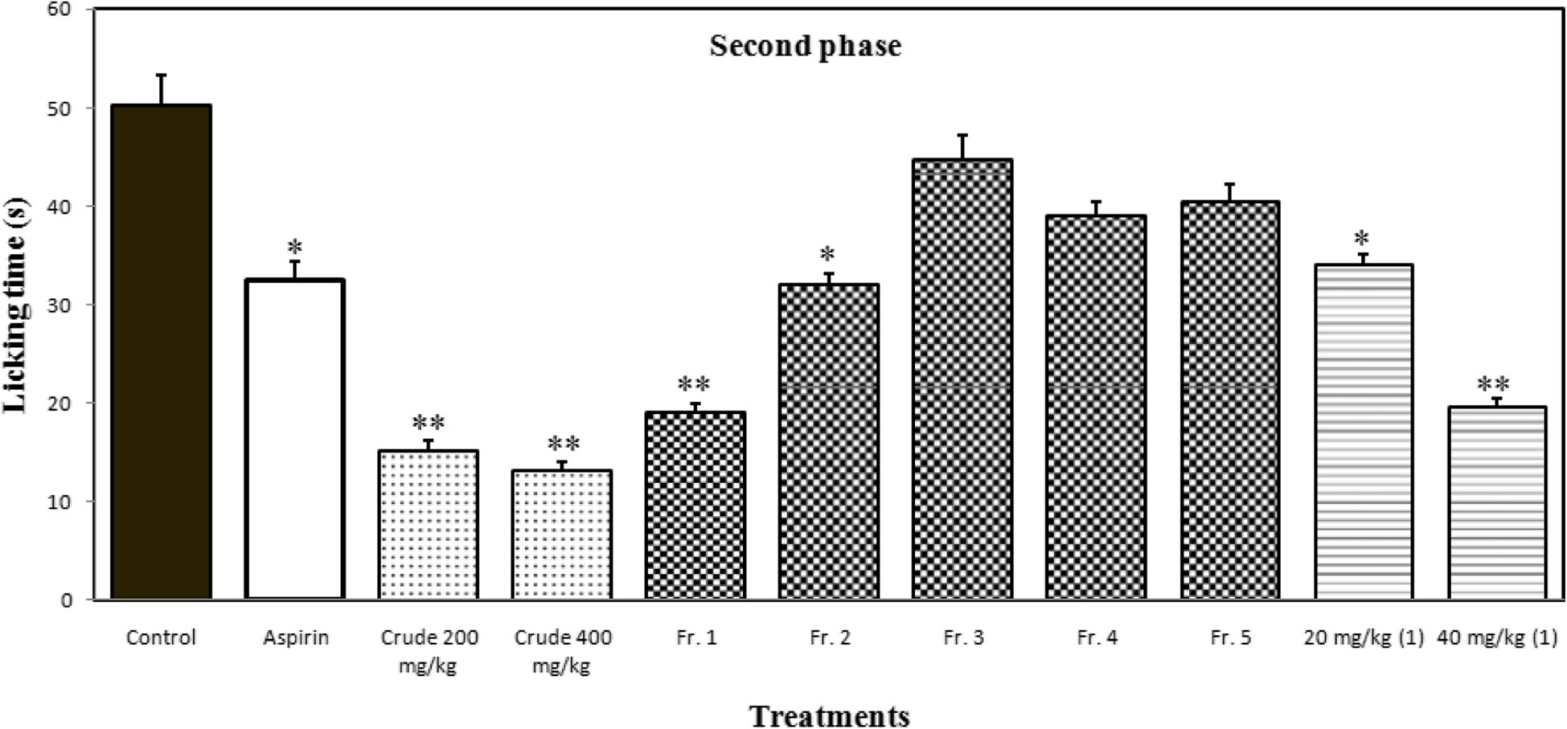
Effects of different doses of MeOH extract, VLC fractions and germacrone (1) on formalin induced licking in mice (Second Phase). Results are mean ± SEM for five mice. Statistical comparison was performed using ANOVA followed by Dunnett’s test. * *p* < 0.05, ** *p* < 0.001, when compared with control group

The MeOH extract showed statistically significant antinociceptive action in both phases compared to the reference drug, aspirin (which exhibited more significant activity in the late phase), by reducing the number of licking at doses of 200 and 400 mg/kg. Thus, the results indicate that the significant pain reduction is might be due to the presence of central and peripheral antinociceptive drugs that are also effective as an anti-inflammatory agents. In this model, aspirin (acetyl salicylic acid, 100 mg/kg) was used to insure the peripheral antinociceptive and possible anti-inflammatory activity because it is more potent in the late phase. The extract used in formalin induced licking exhibited 33.27 and 38.13 % inhibition (*p* < 0.001) in the early phase of licking at doses of 200 and 400 mg/kg, respectively; 69.72 and 73.71 % inhibition (*p* < 0.001) in the late phase of licking at doses of 200 and 400 mg/kg, respectively (Figs. 4 and 5).

The separation of the MeOH extract by VLC eluted 5 fractions (Fr. 1 to Fr. 5). These fractions were screened for the central and peripheral antinociceptive activity using acetic acid induced writhing and formalin induced licking. Fr. 1 was found to have the most activity in both writhing and licking and exhibited 33.98 % (*p* < 0.001) inhibition of acetic acid induced writhing and 23.94 % (*p* < 0.001) inhibition in the early phase and 62.15 % (*p* < 0.001) inhibition in the late phase of formalin induced licking. The result of Fr. 1 was more potent in both methods compared to the other fractions at the same dose of 100 mg/kg.

On the basis of the analgesic activity, Fr. 1 was subjected to a silica gel column chromatography and a total of 68 fractions were obtained. On the basis of the analytical TLC, these fractions were combined into 5 new fractions (SF. 1 to SF. 5) and screened to find the most potent fraction using only acetic acid induced writhing. The fraction SF. 3 exhibited the most potent analgesic activity with inhibition of 46.88 % (*p* < 0.001) in acetic acid induced writhing at a dose of 50 mg/kg that showed most activity than the other new fractions with similar dose (Fig. 3). As both writhing and licking indicated Fr. 1 as the most potent fraction; therefore, we conducted only acetic acid induced writhing for the screening of SF. 1 to SF. 5 to investigate the most potent column fraction that is responsible for the optimum antinociceptive activity of Fr. 1. The antinociceptive principle (2 gm) was purified from the fraction SF. 3 by crystallization with *n*-hexane.

The presence of carbonyl group was shown by the strong absorption in IR spectrum at 1672.4 cm^−1^. The ^1^H-NMR spectrum showed three olefinic proton resonances at *δ* 2.83 (1H, d, *J* = 12.5 Hz), *δ* 2.93 (2H, d, *J* = 11.0 Hz) and *δ* 4.96 (1H, d, *J* = 11.5 Hz). The spectrum also demonstrated six methylene proton peaks at *δ* 2.34 (1H, s, ), *δ* 2.83 (1H, d, *J* = 12.5 Hz), *δ* 2.93 (2H, d, *J* = 11.0 Hz), *δ* 3.39 (1H, d, *J* = 10.1 Hz), *δ* 4.69 (1H, d, *J* = 10.1 Hz) and at *δ* 4.96 (1H, d, *J* = 11.5 Hz), and peaks of four methyl group at *δ* 1.42 (3H, s), *δ* 1.61 (3H, s), *δ* 1.70 (3H, s), and at *δ* 1.75 (3H, s). Based on these spectral data analysis, germacrone **(1)** was identified. Germacrone (**1**) is a cyclic sesquiterpene, which was previously isolated from this plant [10]. ^1^H-NMR data is very consistent with that of germacrone in the literature [26]. It was found to be an active principle that demonstrated 22.66, 34.77 and 51.17 % inhibition (*p* < 0.001) of writhing at doses of 10, 20 and 40 mg/kg, respectively. Thus, the presence activity of acetic acid induced writhing indicates germacrone (**1**) as a peripheral antinociceptive principle of C*. aeruginosa* rhizome*.* Besides, germacrone (**1**) indicated no lethal toxicity in mice at a dose of 750 mg/kg in the literature [23]. The selection of doses of germacrone (**1**) was kept far below the lethal dose 750 mg/kg and was kept consistent with the positive control.

The peripheral antinociceptive activity of germacrone (**1**) by acetic acid induced writhing was also previously investigated by Ozaki (1990) [24] from *C. xanthorrhiza* Roxb, where he determined the activity of germacrone (**1**) in writhing method with 58.08 % inhibition (*p* < 0.01) at a single dose of 75 mg/kg and used indomethacin (10 mg/kg) as a positive control. Moreover, Ozaki (1990) [27] insisted Fr. IX was germacrone (**1**), but did not mention any data or proper citation in support of his claim.

As germacrone (**1)** showed potent peripheral antinociceptive activity in acetic acid induced writhing method, therefore we investigated the effect of germacrone (**1**) in formalin induced licking method to clarify the central antinociceptive effect through both phases and peripheral antinociceptive effect through the second phase. The second phase was also used to investigate the possible effect of drug on inflammation. In formalin induced licking method, germacrone (**1**) showed 30.43 % (*p* < 0.001) and 37.53 % (*p* < 0.001) inhibition in the early phase and 32.27 % (*p* < 0.005) and 60.96 % (*p* < 0.001) inhibition in the late phase at doses of 20 and 40 mg/kg, respectively. The reduction of both phases of licking indicates germacrone (**1**) as a central antinociceptive while the inhibition of the second phase indicates that it is a peripheral antinociceptive drug, which might have possible anti-inflammatory activity. This finding of the possible effect on inflammation strongly support the published literature of anti-inflammatory activity of germacrone (**1**) [13, 27]. Thus, the results indicate the germacrone (**1**) as a centrally and peripherally acting antinociceptive principle of C*. aeruginosa* rhizome with possible anti-inflammatory activity.

## Conclusion

Germacrone (**1**) showed potent activities in both writhing and licking methods indicating the compound is a central and peripheral antinociceptive principle of *C. aeruginosa* rhizome with possible anti-inflammatory activity. Thus, the results also strongly support the literatures of the anti-inflammatory activity of germacrone (**1**). The investigation indicated that *C. aeruginosa* rhizome is a rational choice as folk and traditional medicines for the treatment of rheumatic disorders.

## Abbreviations

VLC: Vacuum liquid chromatography
TLC: Thin layer chromatography
NMR: Nuclear magnetic resonance
FT-IR: Fourier transform infrared
CMC: Carboxymethyl cellulose
SEM: Standard error mean
NSAIDs: Non-steroidal anti-inflammatory drugs

## References

[1] Al-Amin M, Sultana GNN, Hossain CF (2012). Antiulcer principle from *Zingiber montanum*. J Ethnopharmacol.

[2] Hossain CF, Al-Amin M, Rahman KMM, Sarker A, Alam MM, Chowdhury MH, Khan SN, Sultana GNN (2015). Analgesic principle from *Curcuma amada*. J Ethnopharmacol.

[3] Hossain CF, Kin YP, Baerson SR, Zhang L, Bruick RK, Mohammed KA, Agarwal AK, Nagle DG, Zhou YD (2005). *Saururus cernuus* lignans-Potent small molecule inhibitors of hypoxia-inducible factor-1. Biochem Biophys Res Commun.

[4] Thaina P, Tungcharoen P, Wongnawa M, Reanmongkol W, Subhadhirasakul S (2009). Uterine relaxant effects of *Curcuma aeruginosa* Roxb. rhizome extracts. J Ethnopharmacol.

[5] Reanmongkol W, Subhadhirasakul S, Khaisombat N, Fuengnawakit P, Jantasila S, Khamjun A (2006). Investigation the antinociceptive, antipyretic and anti-inflammatory activities of *Curcuma aeruginosa* Roxb. extracts in experimental animals. J Sci Technol.

[6] Philip K, Malek SNA, Sani W, Shin SK, Kumar S, Lai HS, Serm LG, Rahman SNSA (2009). Antimicrobial activity of some medicinal plants from Malaysia. Am J Appl Sci.

[7] Kamazeri TS, Samah OA, Taher M, Susanti D, Qaralleh H (2012). Antimicrobial activity and essential oils of *Curcuma aeruginosa*, *Curcuma mangga*, and *Zingiber cassumunar* from Malaysia. Asian Pac J Trop Med.

[8] Liu Y, Roy SS, Nebie RH, Zhang Y, Nair MG (2013). Functional food quality of *Curcuma caesia, Curcuma zedoaria and Curcuma aeruginosa* endemic to Northeastern India. Plant Foods Hum Nutr.

[9] Moon-ai W, Niyomploy P, Boonsombat R, Sangvanich P, Karnchanatat A (2012). A superoxide dismutase purified from the rhizome of *Curcuma aeruginosa* Roxb. as inhibitor of nitric oxide production in the macrophage-like RAW 264.7 cell line. Appl Biochem Biotechnol.

[10] Suphrom N, Pumthong G, Khorana N, Waranuch N, Limpeanchob N, Ingkaninan K (2012). Anti-androgenic effect of sesquiterpenes isolated from the rhizomes of *Curcuma aeruginosa* Roxb. Fitoterapia.

[11] Takano I, Yasuda I, Takeya I, Itokawa H (1995). Guaiane sesquiterpene lactone from *Curcuma aeruginosa*. Phytochemistry.

[12] Sirat HM, Jamil S, Hussain J (1998). Essential oil of *Curcuma aeruginosa* Roxb. from Malaysia. J Essent Oil Res.

[13] Claeson P, Panthong A, Tuchinda P, Reutrakul V, Kanjanapothi D, Taylor WC, Santisuk T (1993). Three non-phenolic diarylheptanoids with anti-inflammatoryactivity from *Curcuma xanthorrhiza*. Planta Med.

[14] Islam KMN, Rahman ASMH, Al-Mahmud KA (2001). Manual for care and use of laboratory animals.

[15] Ogawa T, Kotani S (1987). Analgesic effects of N-Acetylmuramyl-L-Alanyl-D-Isoglutamine in decreasing the acetic acid-induced abdominal-writhing response. Infect immune.

[16] Udobang JA, Nwafor PA, Okokon JE (2010). Analgesic and antimalarial activities of crude leaf extract and fractions of *Acalypha wilkensiana*. J Ethnopharmacol.

[17] Gene RM, Segura L, Adzet T, Marin E, Inglesias J (1998). *Heterotheca inuloides*:anti inflammatory and analgesic effects. J Ethnopharmacol.

[18] de Sa PGS, Nunes XP, de Lima JT, Filho JAS, Fontana AP, Siqueira JS, Quintans-Júnior LJ, Damasceno PKF, Branco CRC, Branco A, Almeida JRGS (2012). Antinociceptive effect of ethanolic extract of *Selaginella convoluta* in mice. BMC Complement Altern Med.

[19] Seigmund E, Cadmus R, Lu G (1957). A method for evaluating both non-narcoticand narcotic analgesics. Proc Soc of Exp Biol Med.

[20] Gorki F, Correa CR, Filhe VC, Yunes RA, Calixto JB (1993). Potent antinociceptive activity of a hydroalcoholic extract from *Phyllanthus corcovadensis*. J Pharm Pharmacol.

[21] Correa CR, Calixto JB (1993). Evidence of participation of β_1_ and β_2_ kinin receptors in formalin induced nociceptive response in mouse. Br J Pharmacol.

[22] Le Bars D, Gozariu M, Cadden SW (2001). Animal models of nociception. Pharmacol Rev.

[23] Melo MGD, Araújo AAS, Rocha CPL, Almeida EMSA, Siqueira RS, Bonjardim LR, Quintans-Júnior LJ (2008). Purification, physicochemical properties, thermal analysis and antinociceptive effect of atranorin extracted from *Cladina kalbii*. Biol Pharm Bull.

[24] Gorzalczany S, Marrassini C, Mino J, Acevedo C, Ferraro G (2011). Antinociceptive activity of ethanolic extract and isolated compounds of *Urtica circularis*. J Ethnopharmacol.

[25] Su S, Wang T, Duan J, Zhou W, Hua Y, Tang Y, Yu L, Qian D (2011). Anti-inflammatory and analgesic activity of different extracts of *Commiphora myrrha*. J Ethnopharmacol.

[26] Yamazaki M, Maebayashi Y, Iwase N, Kaneko T (1988). Studies on pharmacologically active principles from Indonesian crude drugs. II. hypothermic principle from *Curcuma xanthorrhia* Roxb. Chem Pharm Bull.

[27] Ozaki Y (1990). Anti-inflammatory effect of *Curcuma xanthorrhiza* Roxb. and its active principles. Chem Phrm Bull.

